# Effect of positive end-expiratory pressure and tidal volume on lung injury induced by alveolar instability

**DOI:** 10.1186/cc5695

**Published:** 2007-02-15

**Authors:** Jeffrey M Halter, Jay M Steinberg, Louis A Gatto, Joseph D DiRocco, Lucio A Pavone, Henry J Schiller, Scott Albert, Hsi-Ming Lee, David Carney, Gary F Nieman

**Affiliations:** 1 Department of Surgery, SUNY Upstate Medical University, E Adams St, Syracuse, New York 13210, USA; 2 Department of Biological Sciences, SUNY Cortland, Graham Avenue, Cortland, New York 13045, USA; 3 Department of Surgery, Mayo Clinic, 1st Street SW, Rochester, Minnesota 55905, USA; 4 Department of Oral Biology and Pathology, SUNY Stonybrook, School of Dental Medicine – South Campus, Stonybrook, New York 11794, USA; 5 Savannah Pediatric Surgery Department, Memorial Health University Medical Center, Waters Avenue, Savannah, Georgia 31404, USA

## Abstract

**Introduction:**

One potential mechanism of ventilator-induced lung injury (VILI) is due to shear stresses associated with alveolar instability (recruitment/derecruitment). It has been postulated that the optimal combination of tidal volume (Vt) and positive end-expiratory pressure (PEEP) stabilizes alveoli, thus diminishing recruitment/derecruitment and reducing VILI. In this study we directly visualized the effect of Vt and PEEP on alveolar mechanics and correlated alveolar stability with lung injury.

**Methods:**

*In vivo *microscopy was utilized in a surfactant deactivation porcine ARDS model to observe the effects of Vt and PEEP on alveolar mechanics. In phase I (*n *= 3), nine combinations of Vt and PEEP were evaluated to determine which combination resulted in the most and least alveolar instability. In phase II (*n *= 6), data from phase I were utilized to separate animals into two groups based on the combination of Vt and PEEP that caused the most alveolar stability (high Vt [15 cc/kg] plus low PEEP [5 cmH_2_O]) and least alveolar stability (low Vt [6 cc/kg] and plus PEEP [20 cmH_2_O]). The animals were ventilated for three hours following lung injury, with *in vivo *alveolar stability measured and VILI assessed by lung function, blood gases, morphometrically, and by changes in inflammatory mediators.

**Results:**

High Vt/low PEEP resulted in the most alveolar instability and lung injury, as indicated by lung function and morphometric analysis of lung tissue. Low Vt/high PEEP stabilized alveoli, improved oxygenation, and reduced lung injury. There were no significant differences between groups in plasma or bronchoalveolar lavage cytokines or proteases.

**Conclusion:**

A ventilatory strategy employing high Vt and low PEEP causes alveolar instability, and to our knowledge this is the first study to confirm this finding by direct visualization. These studies demonstrate that low Vt and high PEEP work synergistically to stabilize alveoli, although increased PEEP is more effective at stabilizing alveoli than reduced Vt. In this animal model of ARDS, alveolar instability results in lung injury (VILI) with minimal changes in plasma and bronchoalveolar lavage cytokines and proteases. This suggests that the mechanism of lung injury in the high Vt/low PEEP group was mechanical, not inflammatory in nature.

## Introduction

Acute lung injury and its more severe manifestation, acute respiratory distress syndrome (ARDS), continue to represent significant clinical challenges with daunting mortality rates of up to 60% [1]. Treatment in this patient population remains largely supportive, with mechanical ventilation until the acute insult subsides. Although necessary, positive pressure mechanical ventilation has been implicated as a cause of secondary lung injury, acting to exacerbate and perpetuate the primary lung injury. This ventilator-induced lung injury (VILI) contributes to the high mortality rates associated with ARDS. Three main mechanisms of VILI have been postulated: volutrauma, or alveolar overdistension [2-9]; atelectrauma, or repetitive shear stresses of the alveolar epithelium caused by unstable alveoli recruiting and derecruiting [10,11]; and biotrauma, or inflammation secondary to the mechanical injury induced by volutrauma and atelectrama [12-17].

Protective mechanical ventilation strategies utilizing low tidal volumes (Vts) have become the standard of care in ARDS patients [1,18]. While a recent prospective randomized study with low Vt ventilation found a significant reduction in mortality [18], use of elevated levels of positive end-expiratory pressure (PEEP) has shown promise both in the laboratory [14,19,20] and in a prospective randomized clinical study conducted by Amato and coworkers [21]. However, the relative contributions of low Vt and elevated PEEP to the prevention of VILI remain uncertain and controversial. The effectiveness of low Vt or increased PEEP is presumed to result from a reduction in one or more of the mechanisms of VILI (volutrauma, atelectrauma, and biotrauma), but direct observation of alveoli during mechanical ventilation in a living animal would provide a unique insight into the mechanical stresses on the alveolus; such insight is not possible with other inferential techniques, such as pressure-volume curves, computed tomography scans, and impedance tomography. We use the novel technique of *in vivo *microscopy to observe and measure subpleural alveoli directly and in real time during tidal ventilation in both normal and injured lung.

We hypothesized that reduced Vt and increased PEEP work synergistically to stabilize alveoli, and that stabilizing alveoli lessens VILI. To test these hypotheses, we sought to achieve two goals utilizing two experimental phases: phase I, to identify the combination of Vt and PEEP that produces the most and the least alveolar stability; and phase II, to assess the degree of VILI produced by these two extreme Vt/PEEP combinations.

## Materials and methods

### Surgical preparation

Anesthetized Yorkshire pigs weighing 25–35 kg were pretreated with glycopyrrolate (0.01 mg/kg, intramuscular) 10–15 min before intubation and were pre-anesthetized with telazol (5 mg/kg, intramuscular) and xylazine (2 mg/kg, intramuscular). Sodium pentobarbital (6 mg/kg per hour) was delivered intravenously via a Harvard infusion pump (model 907; Harvard Apparatus, Holliston, MA, USA) to achieve continuous anesthesia. Animals were ventilated using a Galileo™ ventilator (Hamilton Medical, Reno, NV, USA) with baseline ventilation (Vt 12 cc/kg, PEEP 5 cmH_2_O, and fractional inspired oxygen 100%) at a rate of 15 breaths/minute, adjusted to maintain arterial carbon dioxide tension at 35–45 cmH_2_O.

A left carotid artery cutdown was performed to gain access for blood gas measurements (Model ABL 2; Radiometer Inc., Copenhagen, Denmark), blood oxygen content analysis (Model OSM 3; Radiometer Inc.), and systemic arterial blood pressure monitoring. A thermodilution pulmonary artery catheter was inserted through the right femoral vein for mixed venous blood gas and oxygen content sampling, along with cardiac output and lung function determinations (Baxter Explorer™ Baxter Healthcare Corp., Irvine, CA, USA). A triple lumen catheter was placed into the right internal jugular vein for fluid, anesthesia, and drug infusion. Pressures were measured using transducers (Argon™ Model 049-992-000A, CB Sciences Inc., Dover, NH, USA) leveled with the right atrium and recorded on a 16 channel Powerlab/16s (AD Instruments Pty Ltd, Milford, MA, USA) with a computer interface.

### Surfactant deactivation

Surfactant deactivation was achieved by endotracheal instillation with Tween-20 surfactant detergent as previously described [22,23]. Briefly, pigs were placed in the right lateral decubitus position and a 0.75 cc/kg 10% solution of Tween-20 in saline was instilled into the right, dependent lung beyond the tracheal bifurcation. Following lavage, the endotracheal tube was reconnected to the ventilator for three breaths and the lungs were then inflated with a Collins supersyringe to twice the baseline Vt for one breath in order to enhance Tween distribution. The endotracheal tube was suctioned, rendering it free from residual Tween and the previous mechanical ventilation regimen was resumed for several minutes. The animal was then rotated to the left lateral decubitus position, and the Tween lavage procedure was repeated in the left lung.

### *In vivo *microscopy

**Figure 1:**
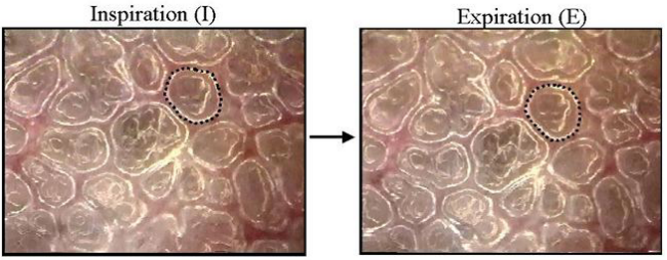
Photomicrographs of the same subpleural alveoli on inflation and deflation. Alveoli of interest are outlined with black dots and depict the same alveolus at expiration and inspiration. Alveolar area at end-expiration (E) was subtracted from the area of the same alveolus at peak inspiration (I) to calculate the degree of alveolar instability (I-EΔ). Note that there is little change in alveolar size in the two dimensions that can be seen using our *in vivo *microscope during tidal ventilation.

A right thoracotomy was performed with removal of ribs five to seven to expose the lung for *in vivo *microscopy. The *in vivo *microscope (epiobjective, epillumination) provides real-time images of subpleural alveoli. Our technique for *in vivo *microscopy is described in detail elsewhere [24] (video footage illustrating the technique is available on the internet [25]). Briefly, the microscope uses a coverslip suction head apparatus. The apparatus is positioned on the visceral pleural surface of the diaphragmatic lobe of the exposed right lung, and gentle suction is applied (5 cmH_2_O) at end-inspiration to affix the lung in place. Suction was minimal to limit motion artifact with respiration, without altering alveolar mechanics [22-24]. The microscopic images were viewed using a video camera (CCD SSC-S20; Sony), recorded using a Super VHS video recorder (SVO-9500 MD; Sony, Tokyo, Japan), and analyzed using a computerized image analysis system (Image Pro™; Media Cybernetics, Carlsbad, CA, USA). Still images of alveoli were extracted from video at peak inspiration and end-expiration, and alveolar areas were measured using computer image analysis (Figure 1). Alveolar stability was expressed as the dynamic change in alveolar area between inspiration and expiration (I-EΔ), with higher values of I-EΔ representative of greater alveolar instability. I-E% was calculated by dividing I-EΔ by the alveolar area at end-expiration.

### Phase I (conducted in three pigs)

Following surgical preparation, continuous filming of subpleural alveoli was performed before surfactant deactivation to serve as controls. Video was recorded during ventilation with all possible permutations of three experimental levels of Vt (6, 12, and 15 cc/kg) and three experimental PEEP levels (5, 10, and 20 cmH_2_O), generating a total of nine experimental groups (Table 1). We chose these tidal volumes because 6 and 12 cc/kg were used in the ARDSnet trial and 15 cc/kg is still used in some hospitals. We felt that the PEEP levels covered the gambit between low, medium, and high PEEP used in current clinical practice. In addition, we chose not to conduct a recruitment maneuver before applying PEEP for two reasons: although recruitment maneuvers are used by many clinicians, they are not currently the standard of care; and it is possible that the recruitment maneuver itself, with a high airway pressure for an extended period of time, could damage the lung [26] and obscure our primary goal of determining the role of multiple ventilator strategies (combination of Vt and PEEP) on alveolar stability and VILI.

**Table 1:** Phase I protocol: alveolar size and stability

		PEEP 5 cmH_2_O	PEEP 10 cmH_2_O	PEEP 20 cmH_2_O
Tidal volume 6 cc/kg				

Control	I	8,820 ± 1,253	9,332 ± 1,229	9,643 ± 1,294
	E	8,677 ± 1,217	9,118 ± 1,236	9,331 ± 1,266
	I-EΔ	142 ± 69	213 ± 46	311 ± 62
	I-E%	1.4 ± 0.8	3.2 ± 0.6	3.7 ± 0.7

Tween	I	12,121 ± 1,184	12,746 ± 1,135*	13,261 ± 1,265
	E	9,293 ± 1,107	11,436 ± 1,008	12,461 ± 1,193
	I-EΔ	2,827 ± 538*	1,310 ± 208*^†^	799 ± 118*^†^
	I-E%	73.5 ± 35	11.3 ± 1.5*^†^	6.3 ± 0.8^a^*^†^

Tidal volume 12 cc/kg				

Control	I	8,888 ± 1,226	9,290 ± 1,247	9,581 ± 1,295
	E	8,719 ± 1,213	9,099 ± 1,228	9,279 ± 1,260
	I-EΔ	169 ± 67	191 ± 81	301 ± 74
	I-E%	2.6 ± 0.7	2.6 ± 1.0	3.3 ± 0.8

Tween	I	12,250 ± 998	13,567 ± 1,093*	13,047 ± 1,307
	E	8,714 ± 1,116	11,927 ± 1,034	13,046 ± 1,307
	I-EΔ	3,535 ± 499*	1,639 ± 155*^†^	1,368 ± 251*^†^
	I-E%	82.8 ± 30.9*	15.3 ± 1.6*^†^	10.9 ± 1.5*^†^

Tidal volume 15 cc/kg				

Control	I	9,143 ± 1,269	9,336 ± 1,242	9,887 ± 1,303
	E	8,936 ± 1,220	9,131 ± 1,233	9,569 ± 1,282
	I-EΔ	207 ± 109	204 ± 60	317 ± 71
	I-E%	2.0 ± 0.9	3.0 ± 0.9	4.1 ± 1.0

Tween	I	14,353 ± 1,224*	14,175 ± 1,169*	14,846 ± 1,518*
	E	8,959 ± 1,201	12,272 ± 1,107	12,911 ± 1,334
	I-EΔ	5,394 ± 750*	1,903 ± 315*^†^	1,934 ± 328*^†^
	I-E%	108.0 ± 32.7^a^*	17.6 ± 2.8*^†^	15.4 ± 2.3*^†^

Shown are alveolar size and stability at all nine combinations of tidal volume (Vt) and positive end-expiratory pressure (PEEP) in both normal lung (control) and acutely injured lung (Tween).

^a^The Vt/PEEP combinations that resulted in the most and least stable alveoli and were used in phase II. E, expiratory alveolar area (μm^2^); I, inspiratory alveolar area; I-EΔ, inspiratory minus expiratory alveolar area (μm^2^); I-E%, % change in alveolar area from peak inspiration to end-expiration ([I - E]/E). *P < 0.05 vs the same Vt and PEEP combination in control lung;^†^*P *< 0.05 versus 5 cmH_2_O PEEP in the Tween group.

The order of the nine combinations was randomized. Ventilation was maintained at each combination for 5 min to acquire video in order to assess alveolar mechanics before changing ventilation. After all nine Vt/PEEP combinations in healthy lung, Tween instillation was performed as described above. The *in vivo *microscope was again placed on the visceral pleural surface and video was recorded for all nine combinations of Vt and PEEP in the surfactant-deactivated lung in a similar manner. It is important to note that the same alveoli were filmed for each Vt/PEEP combination. In the event that alveoli moved out of our field of view for any of the Vt/PEEP combinations, they were excluded from the data analysis. Thus, our data represent the effect of each Vt/PEEP combination on the same individual alveoli in the normal and surfactant-deactivated lung.

The phase I protocol was designed to determine which combination of Vt and PEEP was most effective at stabilizing alveoli. In the subsequent phase II protocol, we tested the hypothesis that the combination of Vt and PEEP determined in the initial phase that resulted in the most stable alveoli would produce the least lung injury, and that the combination that resulted in the most unstable alveoli would result in more severe lung injury. In phase I, we found that a Vt/PEEP combination of 5 cmH_2_O PEEP and 15 cc/kg Vt caused the most alveolar instability (highest I-EΔ and I-E%), and a combination of 20 cmH_2_O PEEP with 6 cc/kg Vt caused the least alveolar instability (lowest I-EΔ and I-E%). Thus, these were the two Vt/PEEP combinations that were tested in phase II.

### Phase II (conducted in six pigs)

Following surgical preparation, the *in vivo *microscope was placed on the visceral pleural surface of healthy swine lung and subpleural alveoli were recorded before Tween instillation to serve as controls. Lavage was then performed with Tween as described above. The *in vivo *microscope was again placed on the visceral pleural surface and animals were divided into two groups: animals in the high Vt/low PEEP group (least alveolar stability) were ventilated with Vt 15 cc/kg and PEEP 5 cmH_2_O; and those in the low Vt/high PEEP group (most alveolar stability) were ventilated with Vt 6 cc/kg and PEEP 20 cmH_2_O. Alveolar size at expiration, inspiration, and the number of alveoli per field were measured at each time point. Five minutes of *in vivo *microscopic footage was recorded every 30 min for three hours. It should be noted that the same four microscopic fields were recorded at each time point to standardize the data collected.

### Histology

At necropsy the lungs were inflated to 25 cmH_2_O pressure and held at this pressure for 60 s to normalize lung volume history. The lungs were than allowed to deflate to atmospheric pressure and the samples were taken immediately as described below. A 3 × 3 × 3 cm cubic section of the right lung taken directly beneath the *in vivo *microscope viewing field and was fixed in 10% formalin. The fixed tissue contained the alveoli that were being observed with the *in vivo *microscope. The tissue was blocked in paraffin and serial sections were made for staining with hematoxylin and eosin.

A blinded observer evaluated lung tissue; details of this scoring methodology are published elsewhere [6]. Briefly, the slides were reviewed at low magnification to exclude areas containing bronchi, connective tissue, large blood vessels, and areas of confluent atelectasis, such that histologic data was from parenchymal tissue. These parenchymal areas were assessed at high magnification (400×) in the following manner. Five high power fields (HPFs) were randomly sampled. Features including alveolar wall thickening, intra-alveolar edema fluid, and number of neutrophils were assessed in each of the five HPFs. Specifically, alveolar wall thickening, defined as greater than two cell layers thick, was graded as '0' (absent) or '1' (present) in each field. Intra-alveolar edema fluid, defined as homogenous or fibrillar proteinaceous staining within the alveoli, was graded as '0' (absent) or '1' (present) in each field. A total score/five HPFs for alveolar wall thickening and intra-alveolar edema fluid was recorded for each animal. The total number of neutrophils was counted in each of the five HPFs and expressed as the total number/five HPFs for each animal. All data are expressed as mean ± standard error.

### Serum/bronchoalveolar lavage fluid cytokines

Serum and bronchoalveolar lavage (BAL) fluid were obtained at baseline and when the animals were killed. Serum and BAL levels (ng/ml) of IL-1, IL-6, IL-8, IL-10, and tumor necrosis factor (TNF)-α were determined by enzyme-linked immunosorbent assay (Endogen, Woburn, MA, USA).

### Neutrophil elastase activity

Neutrophil elastase activity was determined in serum drawn both at baseline and at the end of the experiment, and in BAL fluid obtained at necropsy. Specifically, elastase activity was determined by incubating either 100 μl serum or BAL fluid and 400 μl of 1.25 mmol/l methoxy succinyl-ala-pro-val-p-nitroanilide (specific synthetic elastase substrate) in a 96-well enzyme-linked immunosorbent assay plate at 37°C for 18 hours. After incubation, the optical density was read at 405 nm. Data are expressed as nanomoles elastase substrate degraded per milligram of protein per 18 hours (nmol/l per 18 hours per mg).

### Gelatinase activity

Matrix metalloproteinase (MMP)-2 and MMP-9 activities were measured using a type I gelatin zymography technique. A volume of 20 μl BAL fluid or 2.5 μl serum was electrophoresed (30 mA) for two hours at 4°C. The slab gels were then incubated for one hour with 2.5% Triton X-100 at 22°C and the gels washed with water, then incubated at 37°C in TRIS/NaC/CaCl_2 _buffer overnight. The gels were stained with Coomasie blue, destained with 20% methanol/5% acetic acid (22°C), and the molecular weights of the gelatinolytic zones were compared with standard MMP-2 and MMP-9. The concentrations of MMP-2 and MMP-9 were calculated by scanning of the gels using an image densitometric system (Kodak Image Analysis System; Kodak, Rochester, NY, USA). MMP-2 and MMP-9 concentrations are expressed in densitometric units.

### Lung water

A 2 × 2 × 2 cm section of lung directly adjacent to each histologic section was used for wet-to-dry weight ratio determination. The samples were placed in a dish and weighed, dried in an oven at 65°C for 24 hours, and weighed again. This was repeated until there was no weight change over a 24-hour period, at which time the samples were deemed to be dry. Lung water is expressed as a wet to dry weight ratio.

### Vertebrate animals

The experiments described in this study were performed in adherence with the US National Institutes of Health guidelines for the use of experimental animals in research. The protocol was approved by the Committee for the Humane Use of Animals at our institution.

### Statistical analysis

All values are reported as mean ± standard error. Differences between groups were determined using one-way analysis of variance, and differences within groups were determined using repeated measures analysis of variance. Whenever the F ratio indicated significance, a Newman-Keul test was used to identify individual differences. *P *< 0.05 was considered statistically significant.

## Results

### Combinations of tidal volume and positive end-expiratory pressure

**Figure 2:**
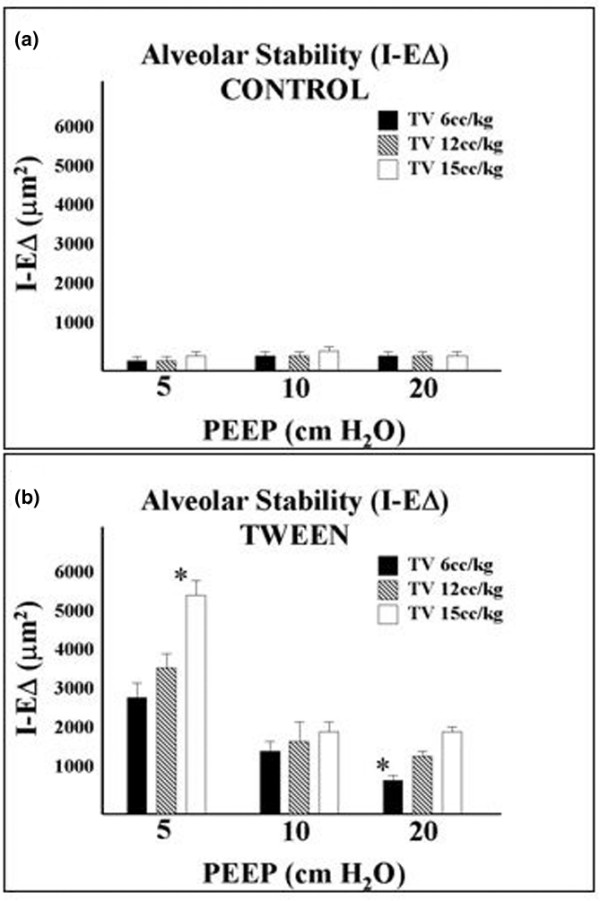
Alveolar stability in the control and Tween-injured lung. In the phase I protocol alveolar stability (I-EΔ) was determined for all nine combinations of tidal volume (Vt) and positive end-expiratory pressure (PEEP). **(a) **Note very stable alveoli (low I-EΔ), regardless of PEEP and Vt, in normal lungs before endotracheal instillation of Tween (Additional file 1). **(b) **After Tween instillation, ventilation with the highest Vt (15 cc/kg) combined with the lowest PEEP (5 cmH_2_O) caused the greatest alveolar instability (highest I-EΔ; Additional file 2), whereas ventilation with the lowest tidal volume (6 cc/kg) and highest PEEP (20 cmH_2_O) resulted in the most stable alveoli (lowest I-EΔ). *The two Vt/PEEP combinations selected for use in the 3-hour ventilator-induced lung injury protocol (phase II).

As expected, control alveoli before Tween endotracheal instillation were very stable during ventilation, with no significant differences for any of the alveolar mechanics parameters (alveolar area at peak inspiration, alveolar area at end-expiration, I-EΔ, and I-E%) regardless of Vt/PEEP combination (Table 1 and Figure 2a; also see Additional file 1). Following Tween endotracheal instillation, significant alveolar instability (high I-EΔ and I-E%) was observed in several Vt/PEEP groups, the most dramatic being the combination of the lowest PEEP (5 cmH_2_O) with the highest tidal volume (15 cc/kg; Table 1 and Figure 2b; also see Additional file 2). For any given tidal volume following Tween instillation, higher levels of PEEP were directly related to alveolar stabilization (lower I-EΔ and I-E%). Furthermore, for any given PEEP setting, progressive increases in Vt produced a progressive trend toward increased alveolar instability (Table 1 and Figure 2b).

### Alveolar stability

**Figure 3:**
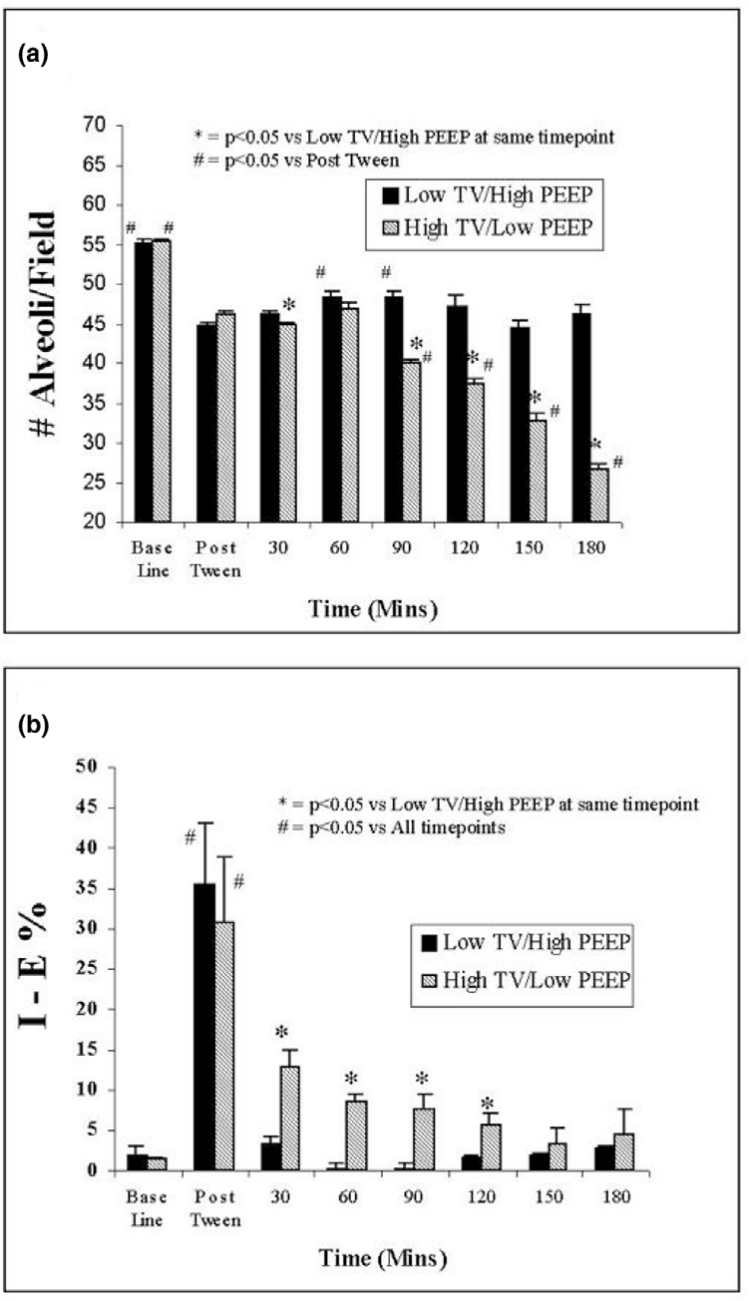
Number of alveoli per microscopic field and alveolar stability over time. In the phase II protocol alveolar microatelectasis and alveolar stability were evaluated. **(a) **Alveolar microatelectasis was measured by counting the number of alveoli per *in vivo *microscopic field; **(b) **alveolar stability was measured as the percentage change in alveolar area from inspiration to expiration (I-E%). Measurements were made before endotracheal instillation of Tween (Baseline), after endotracheal instillation of Tween (Post-Tween), and every 30 min thereafter for 180 minutes. PEEP, positive end-expiratory pressure; Vt, tidal volume.

At baseline before Tween endotracheal instillation, as expected there were no significant differences for any of the alveolar mechanics parameters (alveolar area at peak inspiration, alveolar area at end-expiration, I-EΔ, and I-E%) for either the low Vt/high PEEP or the high Vt/low PEEP group (Figure 3b; also see Additional file 1).

Immediately following Tween instillation alveolar instability increased dramatically, with significantly higher values for I-E% observed for both groups (Figure 3b; also see Additional file 2). In the low Vt/high PEEP group, alveoli were stabilized from the 30 min time point throughout the duration of the three hour study period, with little change in I-EΔ and I-E% values, which were similar to baseline levels (Figure 3b). In contrast, in the high Vt/low PEEP group, alveolar instability persisted as late as two hours into the protocol, with significantly elevated I-E% values compared with baseline levels (Figure 3b).

### Microatelectasis

There were also significant differences in the number of alveoli present in the microscopic field, which we used as a measure of alveolar microatelectasis (Figure 3a). Although both groups demonstrated similar numbers of alveoli at baseline and immediately after Tween instillation, ventilation with the high Vt/low PEEP combination resulted in progressive microatelectasis, because alveoli continually collapsed during the three hour protocol (Figure 3a). In the low Vt/high PEEP group, however, the number of open alveoli remained constant throughout the three hour duration of the study (Figure 3a).

### Hemodynamics and pulmonary parameters

There were no significant differences between the two Vt/PEEP combinations in terms of peak or mean airway pressures, static compliance, and alveolar-arterial gradient at any time point during the 3-hour study (Table 2). Mean airway pressures were statistically higher in the low Vt/high PEEP group. Despite attempts to normalize partial carbon dioxide tension (PCO_2_) with increases in respiratory rate (maximum rate allowed by protocol was 35 breaths/min), hypercapnia in the low Vt/high PEEP group was substantial, resulting in significant respiratory acidosis at all time points compared with the high Vt/low PEEP group. With the exception of oxygen saturation at the 60 and 120 min time points, the low Vt/high PEEP strategy produced superior arterial oxygen tension and oxygen saturation throughout the study (Table 2).

**Table 2:** Phase II protocol: physiologic parameters

		Tween						
		
	Baseline	Instillation	30 min	60 min	90 min	120 min	150 min	180 min
High Vt plus low PEEP								

Ppeak	23 ± 0.6	31 ± 1.3*	37 ± 1.9*^†^	38 ± 2.0*^†^	37 ± 2.0*^†^	37 ± 2.0*^†^	37 ± 2.0*^†^	36 ± 1.9*^†^
Pmean	9 ± 0.4	11 ± 0.1*	12 ± 0.3*	12 ± 1.0*	12 ± 1.0*	12 ± 1.0*	12 ± 1.0*	11 ± 0.3*
Cstat	30 ± 4	18 ± 3*	17 ± 2*	15 ± 3*	18 ± 2*	18 ± 2*	19 ± 2*	19 ± 2*
CO	8.5 ± 1	6.6 ± 1.2	4.9 ± 0.6*	3.7 ± 0.8*^†^	3.3 ± 0.4*^†^	3.4 ± 0.5*^†^	3.4 ± 0.5*^†^	2.7 ± 0.3*^†^
SAT	100 ± 1	80 ± 2.3*	84 ± 1.0*	90 ± 4.2*	91 ± 1.2	92 ± 1.5*	92 ± 0.6*	90 ± 1.0*
PO_2_	295 ± 22	54 ± 5*	53 ± 3*	64 ± 8*	65 ± 5*	70 ± 5*	71 ± 6*	71 ± 4*
PCO_2_	38 ± 0.6	51 ± 2.7*	41 ± 2.0^†^	36 ± 3.2^†^	36 ± 1.9^†^	35 ± 4.1^†^	33 ± 2.5^†^	32 ± 0.6^†^
pH	7.52 ± 0.1	7.42 ± 0.1	7.49 ± 0.1	7.51 ± 0.1	7.53 ± 0.1	7.53 ± 0.1	7.54 ± 0.1	7.54 ± 0.1
Aa	28 ± 13	596 ± 5*	566 ± 40*	578 ± 27*	572 ± 17*	543 ± 25*	520 ± 34*	523 ± 34*

Low Vt/high PEEP								

Peak	23 ± 1.0	33 ± 0.9*	49 ± 0.9*^†^	40 ± 1.8*^†^	42 ± 2.0*^†^	42 ± 2.5*^†^	42 ± 3.1*^†^	43 ± 3.8*^†^
Pmean	10 ± 0.9	12 ± 0.6*	25 ± 0.3^‡^*^†^	24 ± 0.3*^†‡^	25 ± 0.3*^†‡^	25 ± 0.7*^†‡^	25 ± 0.7*^†‡^	26 ± 0.9*^†‡^
Cstat	33 ± 4	19 ± 2*	12 ± 1*^†^	12 ± 1*^†^	12 ± 1*^†^	13 ± 2*^†^	15 ± 2*^†^	15 ± 2*^†^
CO	7.4 ± 1.8	8.9 ± 0.8	8.1 ± 0.8^‡^	6.6 ± 0.8	5.8 ± 1.3	5.0 ± 1.4*	5.9 ± 1.9	5.0 ± 2.0*
SAT	99 ± 0.3	64 ± 3.2^‡ ^*	98 ± 0.3^†‡^	96 ± 1.0^†^	95 ± 0.3^†‡^	88 ± 6.5^†^	98 ± 0.8^†‡^	98 ± 1.0^†‡^
PO_2_	361 ± 115	59 ± 14*	216 ± 62^‡^	178 ± 37^‡^	139 ± 14^‡^	127 ± 10^‡^	138 ± 15^‡^	142 ± 15*^‡^
PCO_2_	49 ± 1.9^‡^	62 ± 0.7^‡^	108 ± 8.9*^†‡^	122 ± 15*^†‡^	119 ± 15*^†‡^	114 ± 13*^†‡^	112 ± 17*^†‡^	103 ± 14*^†‡^
pH	7.41 ± 0.1^‡^	7.30 ± 0.1*^‡^	7.12 ± 0.1*^†‡^	7.07 ± 0.1*^†‡^	7.07 ± 0.1*^†‡^	7.06 ± 0.1*^†‡^	7.05 ± 0.1*^†‡^	7.09 ± 0.1*^†‡^
Aa	53 ± 15	576 ± 14*	362 ± 64*^†^	382 ± 46*^†‡^	425 ± 30*^†‡^	444 ± 27*^†^	463 ± 36*^†^	473 ± 31*^†^

The physiologic parameters recorded were peak airway pressure (Ppeak; cmH_2_O), mean airway pressure (Pmean; cmH_2_O), airway plateau pressure (Pplat; cmH_2_O), static pulmonary compliance (Cstat; ml/cmH_2_O), cardiac output (CO; l/min), hemoglobin oxygen saturation (SAT; %), partial arterial oxygen tension (PO_2_; mmHg), partial arterial carbon dioxide tension (PCO_2_; mmHg), and alveolar arterial oxygen gradient (Aa; mmHg). Data are expressed as mean ± standard error. *P < 0.05 versus baseline;^†^*P *< 0.05 versus post-Tween; ^‡^*P *< 0.05 versus the high Vt/low PEEP group. Vt, tidal volume; PEEP, positive end-expiratory pressure.

### Histology and wet/dry ratio

**Figure 4:**
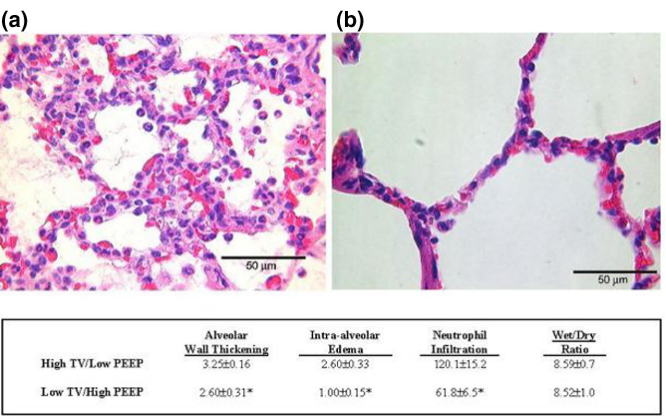
Pathology in the high Vt/low PEEP and low Vt/high PEEP groups. Representative lung histology from the **(a) **high tidal volume (Vt)/low positive end-expiratory pressure (PEEP) and the **(b) **low Vt/high PEEP groups. Morphometric Lung Injury Scores and wet:dry weight ratio are also shown. High Vt/low PEEP caused thickened alveolar walls, numerous neutrophils, and significant intra-alveolar edema. Low Vt/high PEEP ventilation significantly decreased all of the histologic indices of lung injury as compared with the high Vt/low PEEP group. Lung wet:dry weight ratios were not different between groups. Data are expressed as mean ± standard error. *P < 0.05 versus high Vt/Low PEEP group.

Alveolar instability and microatelectasis were associated with a significant lung injury, as measured histologically. High Vt/low PEEP caused alveolar septal thickening, intra-alveolar proteinaceous edema, and neutrophil infiltration. This injury was ameliorated in the low Vt/high PEEP group (Figure 4). There was no difference in lung wet:dry ratio between the two groups (Figure 4). Although there was a significant increase in intra-alveolar edema histologically, the increase was small (Figure 4). Our injury scale is from 0 to 5; the low Vt/high PEEP group scored 1.00 ± 0.15 and the high Vt/low PEEP group scored 2.6 ± 0.33. It is likely that wet:dry ratio was unable to detect such a small difference in intra-alveolar edema.

### Serum and bronchoalveolar lavage cytokines and proteases

Levels of cytokines, MMP-2, MMP-9, and neutrophil elastase for both serum and BAL fluid are reported in Table 3. No significance was identified between the groups in IL-1, IL-6, IL-8, IL-10, TNF-α, MMP-2, or MMP-9 level in either serum or BAL fluid.

**Table 3:** Phase II protocol: cytokine and neutrophil proteases

	Serum baseline	Serum 180 min	BAL fluid
High Vt low PEEP group			

IL-1	0.0 ± 0.0	145.3 ± 72.7	1407.1 ± 338.7
IL-6	126.4 ± 15.5	969.1 ± 321.8	589 ± 146.6
IL-8	10.2 ± 5.9	7.6 ± 3.0	18.0 ± 4.3
IL-10	0.0 ± 0.0	1.3 ± 0.8	2.4 ± 0.9
TNF-α	47.7 ± 29.2	2.8 ± 2.0	9.9 ± 2.4
MMP-2	730.3 ± 41.2	686.0 ± 25.8	705.5 ± 115.5
MMP-9	571.7 ± 91.0	768.2 ± 123.8	1056.8 ± 126.8
NE	91.7 ± 3.9	52.1 ± 7.5*	52.7 ± 14.4

Low Vt high PEEP group			

IL-1	0.0 ± 0.0	157.4 ± 103.8	739.3 ± 60.0
IL-6	63.3 ± 19.9	1736.7 ± 1106.0	662.9 ± 162.6
IL-8	11.8 ± 4.5	14.8 ± 9.0	35.2 ± 11.0
IL-10	0.0 ± 0.0	3.5 ± 2.5	1.9 ± 0.8
TNF-α	195.6 ± 86.7	27.9 ± 18.6	9.7 ± 3.3
MMP-2	877.0 ± 41.9	778.0 ± 103.6	557.3 ± 86.5
MMP-9	627.7 ± 264.7	1315.0 ± 403.9	1240.3 ± 186.0
NE	37.0 ± 1.6^†^	8.9 ± 4.6*^†^	27.3 ± 7.5

Shown are cytokine and neutrophil proteases in serum and bronchoalveolar lavage (BAL) fluid. Intereukin (IL)-1, IL-6, IL-8, IL-10, and tumor necrosis factor (TNF)-α values are expressed as concentration in pg/ml. Concentrations of matrix metalloproteinase (MMP)-2 and MMP-9 are expressed in densitometric units (DU), and neutrophil elastase (NE) as nanomoles of elastase substrate degraded per milligram of protein per 18 hours and expressed as the degradation of substrate over time (nmol/l per 18 hours per mg). Data are expressed as mean ± standard error.*P < 0.05 versus baseline;^†^*P *< 0.05 versus high Vt/low PEEP for same time point. Vt, tidal volume; PEEP, positive end-expiratory pressure.

## Discussion

The most important findings of the present study are as follows: Vt and PEEP act synergistically to stabilize alveoli; increasing PEEP is more effective at stabilizing alveoli than reducing Vt; stabilizing alveoli and preventing microatelectasis with low Vt/high PEEP reduces VILI; and the mechanism of VILI in this three hour animal model appears to be mechanical rather than inflammatory. Ventilating the surfactant-injured lung with high Vt/low PEEP results in a continuum of abnormal alveolar mechanics ranging from slightly unstable alveoli to complete recruitment/derecruitment (Additional file 2). Conversely, ventilation with low Vt/high PEEP stabilizes alveoli and provides an important means of defense against VILI in the setting of abnormal surfactant function. The issues are more complex clinically because the impact of improper mechanical ventilation may vary with the degree of initial lung injury and the heterogeneity of ventilation.

Although low Vt ventilation is not new a concept in protective mechanical ventilation [18], the observations that high PEEP and low Vt work synergistically to stabilize alveoli and that increasing PEEP is more effective than reducing Vt at stabilizing alveoli are unique. If alveolar instability causes lung injury, as both our previous study [27] and present one suggest, it appears that increasing PEEP would provide a greater degree of 'protection' than that provided by reduction in Vt. Examining the trends in I-EΔ when Vt was changed with a similar PEEP reveals that there was a 47.6% decrease in I-EΔ (alveoli were stabilized) between Vt 15 (cc/kg)/PEEP 5 (cmH_2_O) and Vt 6/PEEP 5; a 31.2% decrease between Vt 15/PEEP 10 and Vt 6/PEEP 10; and a 58.7% decrease between Vt 15/PEEP 20 and Vt 6/PEEP 20 (Figure 2b and Table 1). However, I-EΔ decreased to a much greater degree, especially at lower Vt, when PEEP was changed with similar Vt; we saw a 1067% decrease in I-EΔ between Vt 6/PEEP 5 and Vt 6/PEEP 20; a 660% decrease between Vt 12/PEEP 5 and Vt 12/PEEP 20; and a 64.1% decrease between Vt 15/PEEP 5 and Vt 15/PEEP 20. These data demonstrate that PEEP can have a much greater impact on alveolar stabilization than reduced Vt, and they suggest that increasing PEEP may be more beneficial in the prevention of VILI than lowering Vt. In addition, we noted that even when using the ventilator strategy that resulted in the best stabilization of alveoli (low Vt/high PEEP), these alveoli were less stable than normal ones. It is known that unstable alveoli cause VILI [27], but the degree of instability necessary to cause injury is not known. It is possible that the slight increase in instability above normal stability (Figure 2) could be sufficient to cause alveolar damage. If this is true, then other modes of protective ventilation such as high-frequency oscillatory ventilation may cause less VILI than low Vt/high PEEP.

Examination of alveolar mechanics also provides new insight as to the time course of development of VILI. When animals were initially placed on high Vt/low PEEP ventilation, alveoli were unstable compared with those in the low Vt/high PEEP group, but the number of patent alveoli was similar between groups for the first hour (Figure 3a). Mean alveolar stability improved over time in the high Vt/low PEEP group because unstable alveoli progressively derecruited (Figure 3a), suggesting that unstable alveoli will eventually collapse (Figure 3b). Progressive alveolar derecruitment is a concern with low Vt ventilation [19,28-30]; however, progressive derecruitment was also observed with high Vt ventilation in this study. Thus, it appears that with sufficient injury to the alveolus progressive derecruitment can occur even if PEEP is elevated.

### Protection with low tidal volume and elevated positive end-expiratory pressure

Reduced lung injury with low Vt ventilation has been the subject of much investigation, and this strategy has become the standard-of-care for ARDS patients [1,18]. A study by Frank and coworkers [31] demonstrated reduced atelectasis and alveolar epithelial injury when Vt was reduced from 12 to 6 cc/kg. In a clinical trial involving 44 ARDS patients [10], reduction in mean tidal volumes (11.1 versus 7.6 cc/kg) produced a marked reduction in BAL fluid levels of TNF-α, IL-1, IL-6 and IL-8, suggesting that lower Vts may reduce biotrauma-induced VILI.

Although low Vt ventilation has become the standard-of-care for ARDS patients, it may exacerbate lung injury if insufficient PEEP is applied to prevent end-expiratory alveolar collapse [32]. One of the aims of the present study was to show the relative value of lowering Vt versus raising PEEP in reducing alveolar stability. We demonstrated that increasing PEEP from 5 to 10 cmH_2_O with a Vt of 6 cc/kg provided much greater alveolar stability (53.7% decrease in I-EΔ) than reducing Vt from 15 to 6 cc/kg at either 5 cmH_2_O (46.7% decrease) or 10 cmH_2_O (31.2% decrease) PEEP. If these results can be extrapolated to clinical treatment of acute lung injury/ARDS, then there is certainly a clear benefit from low Vt ventilation, but there is a potentially greater benefit from even modest increases in PEEP.

Richard and coworkers [20] demonstrated that alveolar derecruitment is more a function of reduced plateau pressures than of low Vt. In addition, they showed that increased levels of PEEP could prevent derecruitment. These findings are consistent with the results of the present study. In addition, low Vt/high PEEP ventilation – similar to that used in our low Vt/high PEEP group – has yielded improvements in oxygenation [33-35] and reduces both intra-alveolar protein levels [33] and lung injury [34,35], supporting the hypothesis that decreased Vt and increased PEEP work synergistly to reduce alveolar instability and reduce VILI.

### Mechanical trauma versus 'biotrauma'

It has been suggested that injurious mechanical ventilation, such as high Vt and/or low PEEP levels, produces lung injury through biotrauma. Stretch imposed on alveolar epithelial cells has demonstrated dramatic increases in IL-8 release as well as IL-8 gene transcription *in vitro *[13]. A clinical study involving 44 ARDS patients [12] identified a significant reduction in IL-6, IL-8, and TNF in those patients ventilated with a low Vt in combination with elevated PEEP. In the present study histologic injury was significantly worse in the high Vt/low PEEP group, but the levels of inflammatory mediators were not significantly increased by this strategy in either serum or BAL fluid. Furthermore, neutrophil elastase actually declined over time, regardless of ventilation strategy. These data suggest that mechanical trauma (shear stress from unstable alveoli) rather than biotrauma is the initial mechanism of VILI. If this study had been conducted for a longer time, then we hypothesize that inflammatory mediators would have increased in the high Vt/low PEEP group. In our previous study [27] we did identify increases in IL-6 and IL-8 when we extended the study by an additional hour, although proteases were not increased, similar to the present study. There was a significant increase in the number of polymorphonuclear leukocytes in lung tissue in the high Vt/low PEEP group compared with the low Vt/high PEEP group, even though there was no difference in the measured inflammatory mediators. It is known that cytokines are not free floating in the plasma but can be bound to cells. This suggests that there was an increase in the tissue-specific cytokines in lung in the high Vt/low PEEP group that resulted in increased polymorphonuclear leukocyte sequestration. We previously showed in a similar animal model that there can be an increase in tissue bound cytokines (TNF and IL-6) [27].

### Critique of methods

Detailed critiques of this *in vivo *microscopic technique have previously been reported [6,22-24,27,36,37]. This *in vivo *microscopic technique allows measurements of alveoli in only two dimensions, and thus we measured alveolar cross-sectional area at inspiration and expiration and these data were used to calculate changes in alveolar size with ventilation (I-EΔ). Although this technique only measures alveolar mechanics in two dimensions, the mechanics of alveoli in the normal and surfactant-deactivated lung are profoundly different. Therefore, our hypothesis that alveolar instability is injurious to the lung appears valid, despite our inability to measure precise changes in alveolar volume. Additionally, our *in vivo *microscopic technique does not provide us with a global measure of alveolar mechanics, but rather we are restricted to the subpleural alveoli in our microscopic field. We have recently demonstrated that subpleural alveoli do not over-distend even at very high airway pressure (60 cmH_2_O; see the data repository by DiRocco and coworkers [37]), and so we did not expect to observe alveolar over-distension in the PEEP 20/Vt 15 group.

Although not ideal, this technique provides a bridges between purely physiologic approaches to assessment of alveolar mechanics (such as pressure-volume curve analysis) and purely anatomic approaches (such as computed tomography scanning). The short duration of the study might not have been sufficient time to allow a change in inflammatory mediators to take place. Ventilation with low Vt resulted in a significant increase in PCO_2_, which could not be normalized by increasing respiratory rate. It has been shown that high PCO_2 _can protect against VILI [38], and so it is possible that the reduction in tissue injury in the low Vt/high PEEP group could have been due to high PCO_2 _rather than stabilization of alveoli. Finally, we did not use a recruitment maneuver before setting PEEP and Vt, and it is possible that the results of the experiment would have been altered if a recruitment maneuver had been performed.

Although we used a small number of animals in each group (*n *= 3/group), the facts that the data were very tight (low standard error) and that we achieved statistical significance in our primary end-point (alveolar stability) suggest that the study had sufficient power to address the the issue considered in the present study. No bias was introduced by a single animal; otherwise, the data would have had a very high standard error.

In this study we considered whether a combination of Vt and PEEP that resulted in alveolar instability cause lung injury. To address this issue, we directly observed subpleural alveoli for stability and, at necropsy, removed the lung tissue that had been observed using the *in vivo *microscope for histologic analysis. This methodology allowed used to correlate alveolar instability with lung injury and test our hypothesis. However, there were several confounding factors that do not allow us to extrapolate these results readily to the ARDS patient. First, our alveolar sample size was very small and included subpleural alveoli only, so we do not know whether the area of lung sampled is representative of the entire lung. Second, our samples were from nondependent lung areas and our results might have been different in the dependent lung. Finally, we must open the chest to attach the *in vivo *microscope, and so we do not know whether our findings would have been different if we had been able to obtain the same information with a closed chest.

Tween causes serious lung injury, regardless of the type of mechanical ventilation that the Tween-injured lung is subjected to. Static compliance fell significantly in both groups following Tween instillation and did not significantly recover with time. This suggests that the static compliance was at a nadir following Tween and could not be further reduced by VILI. However, we did observe a significant improvement in partial arterial oxygen tension in the low Vt/high PEEP group, suggesting protection of the lung from VILI.

Cytokines were not significantly increased in this study, which is not consistent with many other experiments demonstrating that high Vt/low PEEP ventilation strategies increase plasma and BAL fluid cytokine levels. This could be for two reasons. Tween is a unique injury model, and other studies demonstrating cytokine increase have used other lung injury models. Also, this experiment was very short, and if we had extended the diuration of the study we might have identified significantly increased cytokine levels. Finally, not all studies have demonstrated that cytokines are released with injurious ventilation [39], and our findings support this hypothesis.

## Conclusion

Alveolar instability is one of the primary mechanisms underlying VILI. Within the first three hours of alveolar destabilization, VILI is caused by mechanical (shear stress) and not inflammatory injury. Stabilizing the alveoli with proper ventilator settings significantly reduces VILI. Both lowering Vt and raising PEEP stabilize alveoli, and if applied simultaneously the two act synergistically to prevent alveolar instability. Of the two, increasing PEEP has a more potent stabilizing influence on alveoli than does lowering Vt.

## Key messages

• Protective mechanical ventilation strategies must take into consideration the need to stabilize alveoli in order to prevent VILI.

• Both lowering Vt and increasing PEEP will stabilize alveoli.

• However, the combination of reduced Vt and increased PEEP needed to reduce alveolar instability and prevent VILI optimally has not been determined.

## Abbreviations

ARDS = acute respiratory distress syndrome; BAL = bronchoalveolar lavage; HPF = high-power field; I-EΔ = dynamic change in alveolar area between inspiration and expiration; I-E% = I-EΔ divided by the alveolar area at end-expiration; IL = interleukin; MMP = matrix metalloproteinase; PCO_2 _= partial carbon dioxide tension; PEEP = positive end-expiratory pressure; TNF = tumor necrosis factor; VILI = ventilator-induced lung injury; Vt = tidal volume.

## Competing interests

The authors declare that they have no competing interests.

## Authors' contributions

JMH conducted the experiments, analyzed and graphed the data, and wrote the first draft of the manuscript. JMS assisted JMH in conducting the experiments and edited the manuscript. LAG contributed to the experimental design, data analysis and interpretation, and conducted the histologic analysis. JDD contributed to data analysis and manuscript editing. LAP contributed to data analysis and manuscript editing. HJS contributed to the experimental design of the study, and data analysis and interpretation. SA contributed to manuscript editing. H-ML measured the cytokine and protease concentrations in plasma and BAL fluid. DC contributed to manuscript editing and experimental design. GFN contributed to the design and development of the protocol, data analysis and interpretation, and writing of the manuscript.

## Supplementary Material

Additional File 1 A Quick Time movie file demonstrating the high stability of subpleural alveoli during tidal ventilation in the normal lung. Notice the minimal movement with each breath of the alveoli in the two dimensions that can be seen using our *in vivo *microscopic technique. Each sphere is an individual alveolus. Click here for file

Additional File 2 A Quick Time movie file demonstrating the change in alveolar mechanics (the dynamic change in alveolar size and shape with tidal ventilation) that occur with acute lung injury. The alveoli in this movie were injured by Tween 20 instillation. Tween 20 deactivates pulmonary surfactant and has been used as model of ARDS for several decades. Notice that alveoli are now very unstable, with some alveoli collapsing totally during expiration and than re-expanding during inhalation.
Click here for file
